# Efficacy and tolerability of a monophasic combined oral contraceptive containing nomegestrol acetate and 17β-oestradiol in a 24/4 regimen, in comparison to an oral contraceptive containing ethinylestradiol and drospirenone in a 21/7 regimen

**DOI:** 10.3109/13625187.2011.614029

**Published:** 2011-10-13

**Authors:** Diana Mansour, Carole Verhoeven, Werner Sommer, Edith Weisberg, Surasak Taneepanichskul, Gian Benedetto Melis, Inger Sundström-Poromaa, Tjeerd Korver

**Affiliations:** * Sexual Health Services, Newcastle Hospitals Community Health, Newcastle upon Tyne, UK; † Merck, Oss, Netherlands; ‡ Sydney Centre for Reproductive Health Research FPNSW, Sydney, Australia; § Faculty of Medicine and College of Public Health Sciences, Chulalongkorn University, Bangkok, Thailand; # Department of Obstetrics and Gynaecology, University of Cagliari, Cagliari, Italy; ^ Department of Women's and Children's Health, Uppsala University, Uppsala, Sweden

**Keywords:** Combined oral contraceptive, Nomegestrol acetate, 17β-oestradiol, Efficacy, Safety, Cycle control

## Abstract

**Objectives:**

The primary objective was to assess the efficacy, cycle control and tolerability of a monophasic combined oral contraceptive (COC) containing nomegestrol acetate (NOMAC) and 17β-oestradiol (E2). Effects on acne were evaluated as a secondary objective. Results were compared to those of a COC containing drospirenone (DRSP) and ethinylestradiol (EE).

**Methods:**

Women (aged 18-50 years) were randomised to receive NOMAC/E2 (2.5 mg/1.5 mg) in a 24/4-day regimen (n = 1591) or DRSP/EE (3 mg/30 μg) in a 21/7-day regimen (n = 535) for 13 cycles.

**Results:**

Estimated Pearl Indices for NOMAC/E2 and DRSP/EE were 0.38 and 0.81 in women aged ≤ 35 years and 0.31 and 0.66 for all women (18–50 years), respectively. Scheduled withdrawal bleedings were shorter and lighter among users of NOMAC/E2 and were sometimes absent altogether. Intracyclic bleeding/spotting was infrequent in both groups, and decreased over time. Type and frequency of adverse events were similar to those typically reported for COCs.

**Conclusions:**

These data show that NOMAC/E2 provides high contraceptive efficacy with acceptable cycle control as well as an overall adverse event profile similar to that of DRSP/EE.

## INTRODUCTION

More than half a century since the first combined oral contraceptive (COC) was introduced, over 100 million women use some form of oral contraception^1^. Despite its popularity, the Pill is not without side effects^2^, including serious risk in rare instances^3^. In order to minimise these unwanted effects, COCs have evolved over the years with reductions in the dose of the oestrogen component^4^, development of different progestogens^5^, and modifications in dosing regimens^6^-^8^. While various generations of progestogens have been developed, ethinylestradiol (EE) has persisted as the oestrogen component in nearly all COC formulations, despite direct and indirect evidence of its association with thromboembolic complications^9^,^10^. Early attempts to substitute EE with 17β-oestradiol (E2) were largely unsuccessful predominantly due to poor cycle control^10^-^14^.

Nomegestrol acetate (NOMAC) combined with E2 is a monophasic COC containing a selective progestogen structurally similar to progesterone and E2, an oestrogen that is identical to the endogenously produced oestrogen by women during the menstrual cycle. NOMAC exhibits strong antigonadotropic activity and moderate antiandrogenic properties^15^-^16^, with no oestrogenic, androgenic, glucocorticoid, or mineralocorticoid activity^15^-^18^. In women, the combination of NOMAC and E2 was shown to effectively suppress ovarian function and thus to inhibit ovulation^19^.

In the current study, the contraceptive efficacy, cycle control, and tolerability of the monophasic 24/4 COC containing 2.5 mg NOMAC and 1.5 mg E2 was evaluated in comparison to a monophasic (21/7) COC containing 3 mg drospirenone (DRSP) and 30 μg EE in healthy, fertile women.

## METHODS

### Study design

This was a randomised open-label, comparative multicentre trial of the 2.5 mg NOMAC and 1.5 mg E2 COC versus a COC containing 3 mg DRSP and 30 μg EE (NCT00511199). Trial participants were recruited from gynaecological and/or general practitioner's practices in Europe, Asia and Australia. Doctors were compensated for the costs associated with the time and medical care provided to their patients, while the participating women did not receive financial compensation other than study medicine at no cost and compensation for travelling. On average, each of the 95 centres recruited between 20 and 60 women. Eligible participants who provided written informed consent were randomly allocated in a 3:1 ratio to either NOMAC/E2 or DRSP/EE for 13 consecutive cycles of 28 days, using an interactive voice-response system. The computer-generated randomisation schedule used blocks of four and central allocation in the order of the randomisation call. Due to the broadened age range compared to previous trials with COCs, randomisation was stratified by age group (18—35 years and 36—50 years). The sample size was based on the guidance of the Committee for Medicinal Products for Human Use (CHMP) for the precision of the Pearl Index estimate of an investigational contraceptive drug product^20^.

The study was approved by the Independent Ethics Committee of each trial centre and it was conducted in compliance with current standards and principles of the Declaration of Helsinki and the International Conference on Harmonisation guidelines for Good Clinical Practice.

### Trial participants

Healthy, sexually active women (18—50 years) with a body mass index between 17 and 35 kg/m^2^ who needed contraception and did not plan to use condoms were included if they met none of the exclusion criteria, were willing to provide written informed consent, and were willing to participate in the study for 13 cycles. The most important exclusion criteria entailed contraindications for contraceptive steroids; an abnormal cervical smear (suggesting dysplasia, cervical intraepithelial neoplasia [CIN], SIL, carcinoma in situ, or invasive carcinoma) at screening; a clinically relevant abnormal laboratory result at screening as judged by the investigator; use of an injectable hormonal method of contraception within six months of an injection with a three-month duration, within four months of an injection with a two-month duration, within two months of an injection with a one-month duration; or present use or use within two months prior to the start of the trial medication of the following drugs: phenytoin, barbiturates, primidone, carbamazepine, oxcarbazepine, topiramate, felbamate, rifampicin, nelfinavir, ritonavir, griseofulvin, ketoconazole, sex steroids (except allowed contraceptive methods used before and after the treatment period) and herbal remedies containing *Hypericum perforatum* (St John's Wort).

### Treatment

From day 1 to day 28, one tablet was to be taken orally at approximately the same time daily for 13 consecutive 28-day cycles. Treatment was either with the investigational product (24 tablets containing 2.5 mg NOMAC and 1.5 mg E2 to be taken on days 1-24 and four placebo tablets to be taken on days 25-28) or the comparator (Yasmin : 21 tablets containing 3.0 mg drospirenone and 30 μg EE to be taken on days 1–21 and seven placebo tablets to be taken on days 22–28). Women with no preceding hormonal contraceptive use were instructed to begin with the trial medication on the first day of menstrual bleeding (starting on days 2-5 was allowed if a condom was used too during the first seven days of trial medication use). Those switching from another hormonal contraceptive (COC, vaginal ring, or transdermal patch) started the trial medication anytime within seven days after their last active tablet (in the case of COC use) or preferably on the day of removal, but at the latest when the next application would have been due (for users of the vaginal ring or transdermal patch). Women changing from a progestogen-only pill or implant, or from a hormonal intrauterine system (IUS) were switched immediately after discontinuing their previous method.

A tablet was considered forgotten when taken more than 12 hours late. In such cases subjects were instructed to take the last forgotten tablet as soon as remembered and subsequent tablets as scheduled, even if this would imply taking two tablets on the same day, or at the same time. To maintain contraceptive protection, forgotten tablets might require the additional use of condoms until seven days of uninterrupted daily tablet intake, but this was differently defined for the two treatment groups. Subjects on NOMAC/E2 were allowed to miss one active tablet at any time, or two tablets between days 8 and 17 of a cycle, without the requirement of additional condom use; those on DRSP/EE were only allowed to miss one active tablet in the second week (cycle days 8-14), with all other active tablet omissions requiring additional use of condoms.

### Measurements and analyses

This large multinational trial was designed – in conjunction with an American twin trial of similar size – to obtain a combined 95% confidence interval (CI) for the Pearl Index in women ≤ 35 years of age, which fulfilled the CHMP criterion such that the upper limit of the CI and the point estimate did not exceed 1 with a probability (power) of at least 80%^20^-^21^. A sample size of one third for the comparator drug was considered sufficient to investigate the differences in cycle control, safety and acceptability. These sample size considerations led to 1260 versus 420 subjects for the ≤ 35 year old population. For the overall population (18-50 years) a total sample size of 1560 vs. 520 subjects was planned for NOMAC/E2 and DRSP/ EE, respectively.

All randomised subjects who took at least one dose of trial medication (All Subjects Treated, or AST group) were included in the safety analysis. Contraceptive efficacy analyses were based on the Intent-to-Treat (ITT) group, which consisted of all subjects from the AST group.

Compliance was assessed by counting dispensed tablets and unused tablets and by examining the patient's record in the electronic diaries.

Participants used electronic diaries on a daily basis to record pill intake and to document vaginal bleeding. Condom use and vaginal intercourse information was to be recorded by the woman at the end of each cycle in the electronic diary.

Contraceptive efficacy analyses using the Pearl Index were performed for the ITT group, with the exclusion of ‘not at risk’ cycles, i.e., cycles during which condoms were consistently used, as determined from the electronic diary data. In the event a woman was reported to have become pregnant in a cycle defined as ‘not at risk', the pregnancy would be counted, and the cycle would be included in the exposure (denominator of the Pearl Index). Any exposure recorded after the estimated date of conception was not used in the denominator.

Contraceptive efficacy was assessed by the occurrence of in-treatment pregnancies, i.e., pregnancies with a conception date between the day of the first intake of trial medication and the last day of intake extended by two days (two-day window). The contraceptive efficacy was expressed as the Pearl Index (in-treatment pregnancies per 100 woman-years of exposure) for the ITT group in subjects ≤35 years of age, with the exclusion of cycles (as determined from the electronic diary data) during which condoms were consistently used as a barrier back-up method, and with a woman-year to equal 13 times a cycle of 28 days (=364 days). Additional Pearl Index calculations were performed for the overall age class. The 95% CIs for the Pearl Index were calculated assuming an underlying Poisson distribution^22^. In addition, life-table analyses were performed for the ITT Group excluding backup cycles. This analysis was based on all subjects from the ITT group with at least one cycle without consistent use of condoms. In-treatment pregnancies were by definition the same as used in the Pearl Index calculations. Cumulative pregnancy rates after 13 cycles of treatment (i.e., at day 364) were calculated using Kaplan Meier estimates and 95% CIs, and expressed as rates in 100 women. Life-table analyses were performed in the age class of 18–35 years and the overall age class.

Pearl Index calculations were also done for several subgroups, including age (≤24, 25-35, ≥36 years); race (White, Asian, Black/African American, Other); smoking (yes/no); body weight (< 60, 60-80, >80 kg);BMI (<18.5,18.5-<25,25-<30, ≥ 30 kg/m^2^); and starters/switchers.

Vaginal bleeding was classified as spotting (requiring none or at most one pad/tampon per day) or bleeding (requiring more than one pad/tampon per day). A reference period (RP) analysis was performed in accordance with World Health Organisation (WHO)^23^-^24^ and CHMP recommendations. This analysis used a fixed 91-day reference period as the basis and was performed for the ITT Group. Reference periods were considered evaluable if no more than two consecutive days with missing bleeding information occurred within a RP. There were four RPs: RP1 from day 1 to day 91; RP2 from day 92 to day 182; RP3 from day 183 to day 273; RP4 from day 274 to day 364 (the theoretical end of the trial).

The vaginal bleeding patterns were also analysed by a so-called cycle analysis based on the bleeding records from the electronic diary. This analysis used evaluable cycles as the basis and was performed for the ITT group. Cycles were defined as evaluable if they had a length of between 22 and 35 days, and no more than two consecutive days with missing bleeding information. One or two consecutive days with missing bleeding information were interpolated by the bleeding information of the preceding day; the interpolation rule was therefore the same as in the RP analysis.

For subjects in the DRSP/EE group, a 28-day cycle consisted of an ‘expected non-bleeding period’ of 21 days during active tablet intake followed by a seven-day ‘expected bleeding period’ of placebo tablets starting on day 22. Because NOMAC/E2 is given in a 24/4 regimen, the ‘expected bleeding period’ was adjusted to the same length of seven days in order to allow comparisons between the two treatment groups; for NOMAC/E2, the ‘expected bleeding period’ started on the day of the first placebo tablet (day 25) and ended on day 3 of the next cycle. As a result, the ‘expected non-bleeding period’ in the NOMAC/E2 group started on day 4 of active pill intake.

The two primary vaginal bleeding parameters in the cycle analysis were (i) the occurrence of breakthrough bleeding/spotting, and (ii) the absence of withdrawal bleeding. Withdrawal bleeding was classified as any bleeding/spotting episode that began or continued into the ‘expected bleeding period'. Breakthrough bleeding/spotting was classified as any bleeding/spotting episode that occurred during the ‘expected non-bleeding period', unless already classified as withdrawal bleeding.

For both primary bleeding parameters, exact binomial, 2-sided 95% CIs were calculated per treatment group and cycle. Treatment groups were compared for cycles 2–13 using 2-sided 95% CIs (normal approximation) for the differences between the groups. No comparisons were done for cycle 1 due to different starting procedures related to pre-trial use of contraceptives. Data on withdrawal bleeding in cycle 13 were incomplete and are not presented (e.g., subjects shortened the last placebo period, or did not report the complete bleeding data for the full length of the 'expected bleeding period’ at the end of cycle 13 and the beginning of the subsequent follow-up cycle).

Secondary vaginal bleeding parameters in the cycle analysis included: the occurrence of breakthrough bleeding, the occurrence of breakthrough spotting (spotting only), the number of breakthrough bleeding/ spotting days, and the number of withdrawal bleeding/ spotting days.

The effects on acne were also evaluated, as a secondary objective. Regular skin examinations were carried out during the trial in order to explore the effect of both COCs on acne. Acne was examined by the study staff at screening and at all visits after randomisation and they were asked to score acne as being ‘none', ‘mild', ‘moderate’ or ‘severe’ according to their own judgment. The study staff recorded any increase in severity of acne from baseline on the adverse events’ (AEs) form.

Safety data were obtained by monitoring of (serious) AEs, routine laboratory parameters, vital signs and cervical smear, and by performing physical and gynaecological examinations.

All statistical analyses were done using SAS statistical software for Windows (SAS Institute, Cary NC, USA).

## RESULTS

### Subjects

**Figure 1:**
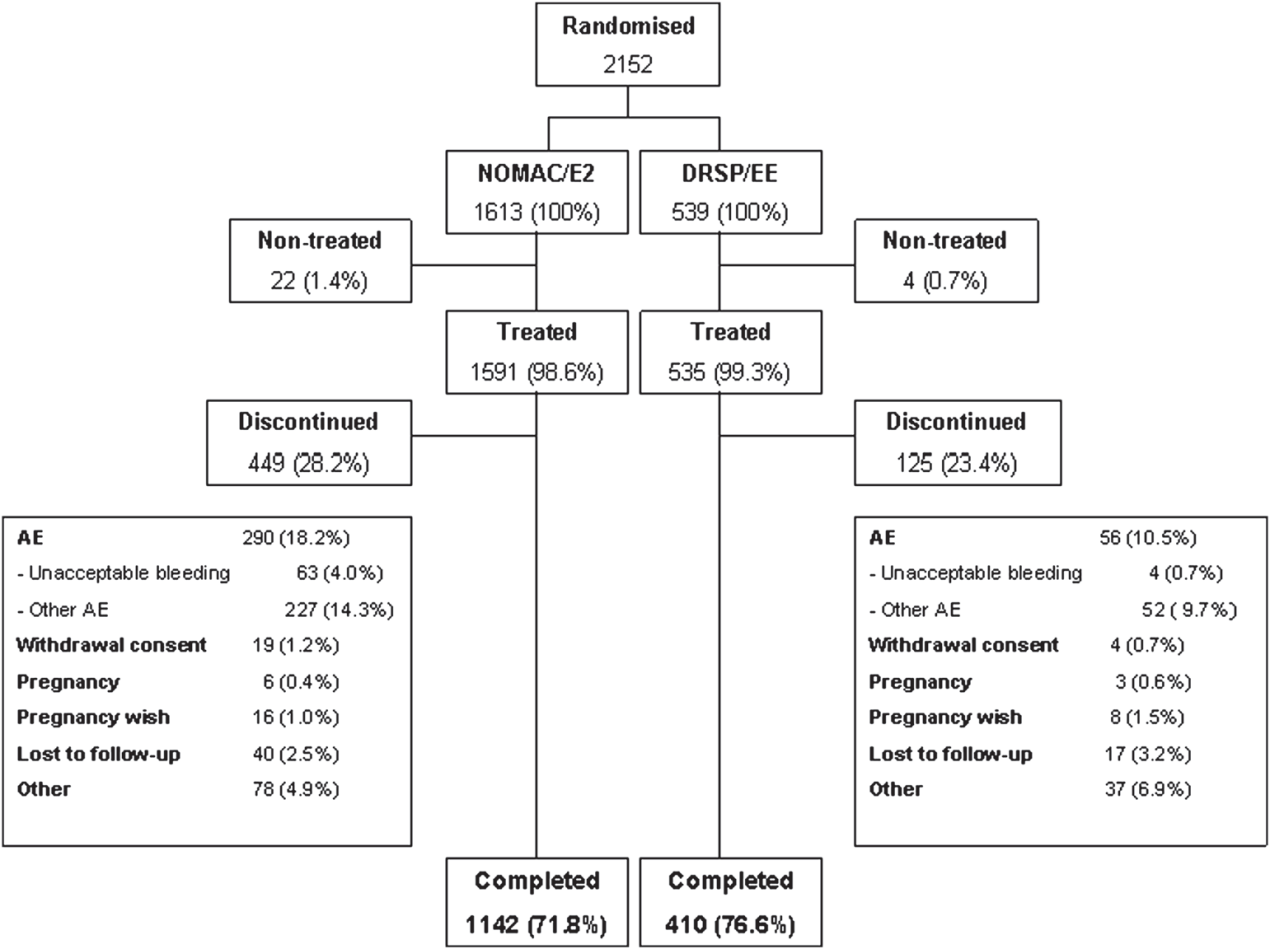
Patient flow through the trial. NO MAC, nomegestrol acetate; E2, oestradiol; EE, ethinylestradiol; DRSP, drospirenone; AE, adverse event.

Out of a total of 2152 women, 1613 were randomised to NOMAC/E2 and 539 to DRSP/EE. Of the 2152 randomised women, 2126 were treated and 1552 completed the trial. In the NOMAC/E2 group, 22 subjects were not treated due to withdrawal of consent (*n* = 5), pregnancy (*n* = 8), and other personal reasons (*n* = 9). Of 1591 treated women, 449 (28.2%) discontinued prematurely. In the DRSP/EE group, four subjects were not treated due to withdrawal of consent (*n* = 1), pregnancy (*n* = 1) and other personal reasons (*n* = 2). Out of 535 women treated, 125 (23.4%) discontinued prematurely (Figure 1). Demographic characteristics of participants were similar in both groups (Table 1).

**Table 1:** Demographic characteristics of subjects at screening.

	*NOMAC/E2*	*DRSP/EE*	*Total*
N	1591	535	2126
Age			
Mean years (SD)	28(7)	28 (7)	28 (7)
18-35 years *n* (%)	1319 (82.9%)	443 (82.8%)	1762 (82.9%)
36-50 years *n* (%)	272 (171%)	92 (172%)	364 (171%)
Race n (%)			
Asian	77 (4.8%)	26 (4.9%)	103 (4.8%)
Black/African American	4 (0.3%)	3 (0.6%)	7 (0.3%)
White/Caucasian	1501 (94.3%)	501 (93.6%)	2002 (94.2%)
Other	9 (0.6%)	5 (0.9%)	14 (0.7%)
Ethnicity n (%)^14^			
Missing	1	0	1
Hispanic or Latino	15 (0.9%)	8 (1.5%)	23 (1.1%)
Not Hispanic or Latino	1575 (99.1%)	527 (98.5%)	2102 (98.9%)
Body weight, mean kg (SD)	63.4 (10.5)	63.7 (10.1)	63.5 (10.4)
BMI, mean kg/m^2^ (SD)	23.0 (3.5)	23.0 (3.4)	23.0 (3.5)
Smoking *n (%)*	381 (24.0%)	146 (273%)	527 (24.8%)
Contraceptive method at screening *n (%)*			
None	155 (9.7%)	58 (10.8%)	213 (10.0%)
COC			
All	1072 (674%)	356 (66.5%)	1428 (672%)
DRSP/EE only	281 (177%)	73 (13.6%)	354 (16.7%)
Foam, condom, suppositories, diaphragm	255 (16.0%)	83 (15.5%)	338 (15.9%)
POP	34 (2.1%)	16 (3.0%)	50 (2.4%)
Hormonal ILJD	10 (0.6%)	5 (0.9%)	15 (0.7%)
Non-hormonal ILJD	14 (0.9%)	6 (1.1%)	20 (0.9%)
NuvaRing®	28 (1.8%)	5 (0.9%)	33 (1.6%)
Patch	6 (0.4%)	4 (0.7%)	10 (0.5%)

NOMAC/E2, nomegestrol acetate/17β-oestradiol (NOMAC/E2); DRSP/EE, drospirenone/ethinylestradiol (DRSP/EE); BMI, body mass index; COC, combined oral contraceptive; POP progestogen-only pill; ILJD, intrauterine device; POP progestogen-only pill; SD, standard deviation.

### Compliance

Based on data of returned tablets, compliance was high, with 94.8% and 91.4% of women in the NOMAC/E2 and DRSP/EE groups, respectively, taking the daily tablet on at least 95% of the days during the treatment period. A similar compliance was calculated on the basis of tablet intake recorded in the daily diary; based on non-missing intake data, 94.9% of NOMAC/E2 users and 91.4% of DRSP/ EE users took the daily tablet on at least 95% of the treatment days.

### Efficacy

The primary efficacy analysis (Table 2) was based on in-treatment pregnancies among women ≤ 35 years in cycles considered at risk of pregnancy (i.e., excluding cycles during which condoms were always used). In the NOMAC/E2 group four pregnancies occurred during 1058 woman-years of exposure and the corresponding Pearl Index estimate in women ≤ 35 years was 0.38 (95% CI 0.10-0.97). In one case the pregnancy occurred in an apparently treatment-compliant woman, without conditions or concomitant drug intake that might interfere with contraceptive efficacy; in the other three cases, circumstances possibly affecting contraceptive efficacy were noted, namely, an earlier period of diarrhoea, an earlier bout of severe vomiting and one case with suggested, but insufficiently documented, non-compliance with tablet intake. In the DRSP/EE group, three in-treatment pregnancies occurred during 372 woman-years of exposure, resulting in a Pearl Index of 0.81 (95% CI 0.17-2.35) in women ≤ 35 years. In one case, non-compliance with tablet intake was confirmed, in the other two cases, non-compliance was suspected but insufficiently documented. Overall, in the same age group, 3.1% of the cycles on NOMAC/E2 and DRSP/EE were affected by documented insufficient compliance, i.e., use of prohibited concomitant medication, missing more than four active tablets in a cycle, or more than two active tablets in a row. For users ≤ 35 years old, results from the subgroup analysis suggested that the contraceptive efficacy of NOMAC/E2 was independent of the demographics and subgroups analysed (age, race, smoking status, weight and BMI; results not shown). In the age group 36–50 years no pregnancies occurred. Life-table analyses were consistent with the Pearl Index estimates, with cumulative one-year pregnancy rates in women ≤ 35 years of 0.40 (95% CI 0.15-1.06) and 0.77 (95% CI 0.25-2.39) for NOMAC/E2 and DRSP/EE, respectively (Table 2).

**Table 2:** Summary of contraceptive efficacy (Pearl Index and life-table analysis) for nomegestrol acetate/17β-oestradiol (N0MAC/E2) and drospirenone/ethinylestradiol (DRSP/EE) in the overall population and age subgroups.

	*NOMAC/E2*		*DRSP/EE*	
		
	*18-50 years*	≤ *35 years*	*18-50 years*	≤ *35 years*
N	1587	1315	534	442
Pearl Index analysis				
Exposure (woman-years)	1292.5	10576	452.8	372.4
Pregnancies*	4	4	3	3
Pearl Index estimate	0.31	0.38	0.66	0.81
95% Cl	0.08, 0.79	0.10, 0.97	0.14, 1.94	0.17, 2.35
Life-table analysis				
Cumulative pregnancy rate after 1 year (%)	0.33	0.40	0.64	0.77
95% Cl	0.12, 0.87	0.15, 1.06	0.21, 1.97	0.25, 2.39

Cl, confidence interval.

* Pregnancies with conception date from the day of first intake of trial medication up to and including the days of last intake of trial medication extended with a maximum of two days. This Pearl Index was calculated by excluding cycles with condom use from the analysis.

Thus, the pregnancy rates for NOMAC/E2 were consistently lower than those for DRSP/EE, but the differences between the treatment groups were not statistically significant.

### Vaginal bleeding

**Figure 2:**
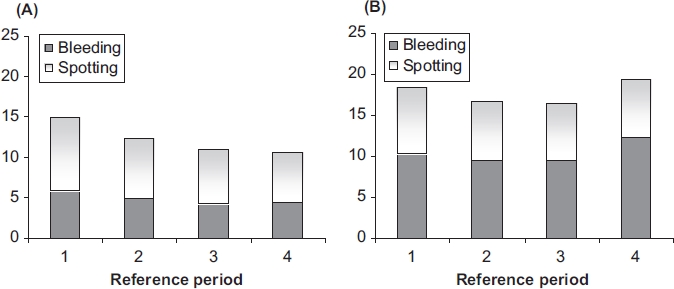
Mean number of bleeding-spotting days per 91-day reference periods for (A) NOMAC/E2 and (B) DRSP/EE. NOMAC, nomegestrol acetate; E2, oestradiol; EE, ethinylestradiol; DRSP drospirenone.

The data of the RP analysis showed a lower mean number of bleeding/spotting days in the NOMAC/ E2 group compared with the DRSP/EE group across the reference periods (Figure 2). For NOMAC/E2 the number of bleeding-spotting days per reference period declined from 14.9 during RP1 to 10.6 during RP4, while for DRSP/EE the numbers remained the same over time, i.e., 18.5 (RP1) and 19.2 (RP4). The difference between the two treatments increased with time to about 8.6 days per reference period at RP4, and was largely caused by an excess of bleeding days with DRSP/EE as compared to NOMAC/E2 (12.4 vs. 4.4 days in RP4). According to the definitions of the reference period analysis, the incidence of amenorrhoea, i.e., no bleeding-spotting at all over a consecutive period of 91 days, increased from 8.1% in RP2 to 13.4% in RP4 for NOMAC/E2 users; amenorrhoea was almost absent among users of DRSP/EE (1.1% in RP4).

**Figure 3:**
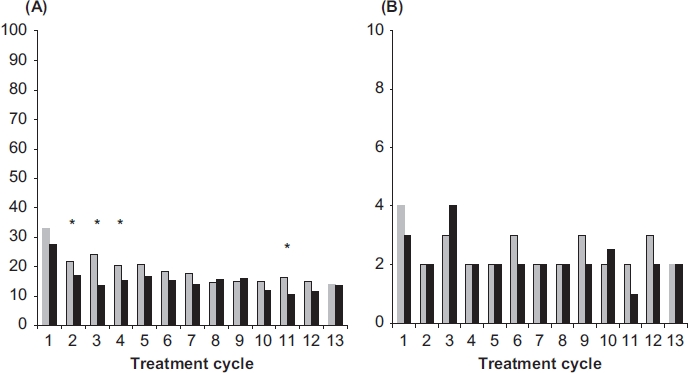
(A) Incidences (%) and (B) duration (median number of days) of breakthrough bleeding-spotting for N0MAC/E2 (grey bars) and DRSP/EE (black bars). The incidences were compared statistically between the treatment groups. *p < 0.05 vs. DRSP/EE. NOMAC, nomegestrol acetate; E2, oestradiol; EE, ethinylestradiol; DRSP drospirenone.

In the cycle analysis breakthrough bleeding/spotting was found to progressively decrease in both groups over the course of the trial. The respective incidences for NOMAC/E2 and DRSP/EE (Cycles 4-13) were similar and ranged from 20% to 14% and from 17% to 11%, respectively, and occasionally reached statistical significance between treatments (Figure 3A). For women with breakthrough bleeding/spotting, the median number of days per cycle was similar between treatment arms (2-3 days in the NOMAC/E2 group and 1-4 days in the DRSP/EE group) (Figure 3B). In both treatment groups, the majority (> 75%) of breakthrough bleeding/spotting episodes consisted of spotting only.

**Figure 4:**
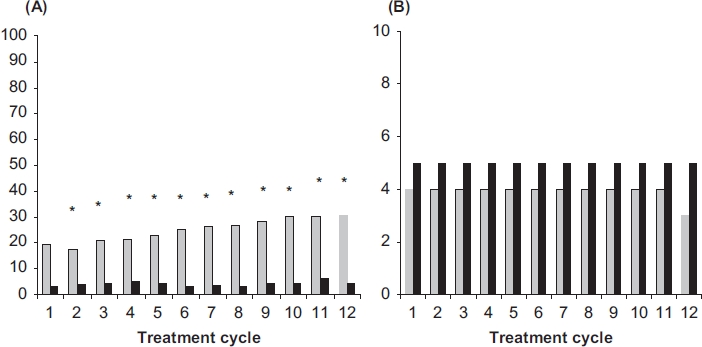
(A) Incidences of absence of withdrawal bleeding (%) and (B) duration of withdrawal bleeding (median number of days) for NOMAC/E2 (grey bars) and DRSP/EE (black bars). The incidences were compared statistically between the treatment groups. *p < 0.05 vs. DRSP/EE. NOMAC, nomegestrol acetate; E2, oestradiol; EE, ethinylestradiol; DRSP drospirenone.

Scheduled withdrawal bleedings were shorter and lighter among users of NOMAC/E2, and were sometimes absent altogether. A progressive increase in the incidence of absence of withdrawal bleeding was observed in the NOMAC/E2 group, ranging from 22% (Cycle 4) to 31% (Cycle 12), which was not observed in the DRSP/EE group (varying between 3 and 6% without particular trend). The difference between the treatment groups was statistically significant for all cycles (Figure 4A). For women experiencing withdrawal bleeding, the median number of withdrawal bleeding/spotting days was lower when using NOMAC/E2 (range 3–4 days) compared to DRSP/EE (5 days) (Figure 4B), the difference being caused by the median number of bleeding days, i.e., two days for NOMAC/E2 and three days for DRSP/EE. Calculated over the entire trial period for all NOMAC/E2 users and based on evaluable cycles, 43% of women on NOMAC/E2 never missed a withdrawal bleeding, while another 21% missed exactly one, and another 9% missed two withdrawal bleedings.

To investigate the predictability of absence of withdrawal bleeding, additional analyses were performed on the subgroup of women who had missed their withdrawal bleeding in at least one of the cycles 2, 3 or 4. This subgroup comprised 30% of the women enrolled on NOMAC/E2 and the incidence of absence of withdrawal bleeding during later cycles (cycles 5-13) ranged between 53% and 66%. This increased tendency for absent withdrawal bleeding was also noted in the similarly defined subgroup of the DRSP/ EE group, although the number of women qualifying for the subgroup was too low to generalise results. The incidence of breakthrough bleeding-spotting during cycles 5–13 among women who experienced absence of withdrawal bleeding during any of the cycles 2, 3 or 4 ranged between 11% and 23%, and was comparable to the overall group incidence (14–21%);absence of withdrawal bleeding in a given cycle was not associated with the occurrence of breakthrough bleeding-spotting in the next cycle, nor was the occurrence of breakthrough bleeding-spotting in a given cycle correlated with absence of withdrawal bleeding in the subsequent expected bleeding period.

### Acne

**Figure 5:**
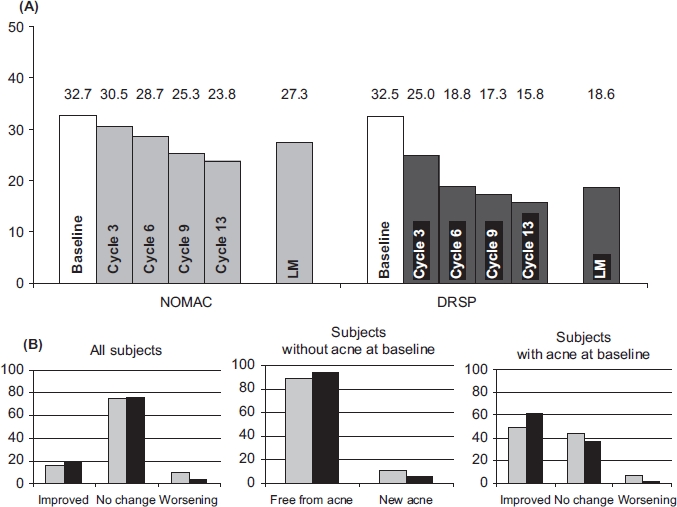
(A) Prevalence (%) of acne and (B) changes in acne severity (%) from baseline to last measurement during treatment with NOMAC/E2 (grey bars) and DRSP/EE (black bars). NOMAC, nomegestrol acetate; E2, oestradiol; EE, ethinylestradiol; DRSP drospirenone; LM, last measurement.

Acne was actively assessed by the study staff at screening and at all visits after randomisation. At baseline, acne was present in 32.7% and 32.5% of women assigned to the NOMAC/E2 and DRSP/EE group, respectively. Overall, the presence of acne compared to baseline decreased over time with both treatments (Figure 5A). With respect to individual changes (Figure 5B), the majority of women (∼75%) in both groups showed no change in acne status at last measurement. For NOMAC/E2 and DRSP/EE, respectively, improvement was observed in 15.9% and 20.1% of participants, and worsening or development of new acne were observed in a smaller group of 9.9% and 4.0%, respectively. Of those presenting with acne at baseline – approximately one third of all women – improvement was noted in 48.4% (NOMAC/E2) and 61.4% (DRSP/EE) of women, while worsening was judged to have occurred in 7.2% and 1.8% of the NOMAC/E2 and DRSP/EE subjects, respectively. Of the women presenting without acne at baseline, the vast majority remained free of acne; in 11.1% (NOMAC/E2) and 5.1% (DRSP/EE) of the women, newly developed acne was observed at last measurement.

### Safety and tolerability

Approximately 80% of all participants reported one or more AE during the in-treatment period. In the NOMAC/E2 and DRSP/EE groups, 51.2% and 37.0% of the women, respectively had AEs that were determined by the investigator to be treatment-related. The most frequently reported treatment-related AEs (related incidence ≥ 5%) were ‘acne’ (15.3% for NOMAC/E2 vs. 7.1% for DRSP/EE); ‘withdrawal bleeding irregular’ (11.7% vs. 0.4%);'weight increased’ (7.9% vs. 6.2%); and ‘headache’ (6.6% vs. 6.2%). Three of these AEs accounted for most of the difference noted in the discontinuation rates due to AEs (Figure 1), with respective discontinuation rates for NOMAC/ E2 and DRSP/EE of 3.3% vs. 0.2% for acne, 4.0% vs. 0.7% for irregular (withdrawal) bleeding, and 1.4% vs. 0.7% for weight increase. Discontinuation owing to irregular bleeding did not occur early in the trial, but rather gradually over time in the NOMAC/E2 group. Serious AEs were reported for 2% of subjects, which were evenly distributed between the two treatment groups. Three SAEs were deemed as (possibly) treatment-related by the investigators, one in the NOMAC/ E2 group ('severe menorrhagia') and two in the DRSP/EE group ('deep vein thrombosis left calf and 'systemic lupus erythematosus with concomitant patellar tendon bearing').

Routine laboratory and blood pressure measurements showed no remarkable changes in mean or median values from baseline in either treatment group. Women on NOMAC/E2 experienced a small increase in mean body weight, i.e., from 63.4 kg at baseline to 64.4 kg at last measurement; women on DRSP/EE increased from 63.7 kg at baseline to 64.0 kg at last measurement. The change from baseline in weight at last measurement was statistically significant between NOMAC/E2 and DRSP/EE (*p* = 0.001).

## DISCUSSION

Results of this study show that the new monophasic COC containing the progesterone-derived progestogen NOMAC in combination with the natural oestrogen E2 provides robust contraceptive efficacy, acceptable cycle control, and shorter, lighter periods. This new 24/4 regimen COC was well tolerated with a safety profile similar to that of DRSP/EE.

Contraceptive efficacy as expressed via the estimated Pearl Indices (95% CI) for women :≤ 35 years were 0.38 (0.10-0.97) for NOMAC/E2 and 0.81 (0.17-2.35) for DRSP/EE. These values represent the first estimation of the contraceptive efficacy for NOMAC/E2. The values determined for DRSP/EE are similar to those previously reported for this COC in a European study population^25^. For NOMAC/E2 users, the contraceptive efficacy appeared to be independent of the demographics (age, race, smoking status, weight and BMI), indicating the strong contraceptive efficacy of NOMAC/E2. Moreover, the contraceptive efficacy in the present study was achieved despite applying less stringent barrier method (condom) back-up contraception requirements in users of NOMAC/E2 when tablets had been missed. NOMAC/E2 users were allowed to miss one active tablet any time and two active tablets mid-cycle (Day 8–17) without back-up requirement, while for DRSP/EE users, one missed active tablet during the first or third week, or two or more active tablets at any time, required the use of back-up contraception. This high contraceptive efficacy of NOMAC/E2 is consistent with the profound ovarian suppression observed with NOMAC/E2^19^. The shorter hormone-free interval^26^-^28^ of four versus seven days for the DRSP/EE COC, combined with the longer terminal elimination half-life of NOMAC^18^,^29^-^30^ compared to DRSP may also have contributed to the numerically lower Pearl Index values in the NOMAC/E2 group, as observed in this study.

NOMAC/E2 is associated with a vaginal bleeding pattern characterised by shorter and lighter bleeding episodes as compared to DRSP/EE. The total number of bleeding-spotting days over 91-day reference periods declined for NOMAC/E2 users over Reference Period 1 to 4 from 14.9 to 10.6; for DRSP/EE users the respective numbers were 18.5 and 19.2, the excess being due to bleeding days.

While the incidence and length of breakthrough bleeding-spotting episodes were largely comparable between the treatment groups, absence of withdrawal bleeding was greater in NOMAC/E2 users (22-31% of NOMAC/E2 users experienced absence of withdrawal bleeding during Cycles 4-12). For NOMAC/ E2 users who had a withdrawal bleed, it was in comparison to DRSP/EE of shorter duration (3-4 days vs. 5 days) and of lighter intensity (2 vs. 3 bleeding days). Missed withdrawal bleeding may be an inherent feature of the shorter hormone-free period^7^,^31^,^32^. Missed withdrawal bleedings with NOMAC/E2 may be reinforced by the particularly long elimination half-life of NOMAC^18^,^29^-^30^. Although absence of withdrawal bleeding has become widely accepted^33^, it was not anticipated at the start of the current trial. Consequently, doctors and women were not counselled beforehand on this aspect and this may have contributed to a somewhat lower acceptance of the bleeding pattern (as reflected in the AE and discontinuation data), particularly in view of fear of unintended pregnancy. In fact the latter should not be of concern given the extent of ovarian suppression^19^ combined with the NOMAC/E2 contraceptive efficacy data from this study. Adequate counselling on the relative paucity of vaginal bleeding and the high contraceptive efficacy will likely result in user satisfaction with NOMAC/E2.

From the data it also appears that NOMAC/E2 prevents intracyclic bleeding as effectively as the DRSP/EE comparator, which contains 30 μgEE. This is a remarkable observation in view of the initial results with COCs containing E2, which showed inadequate cycle control that prevented their successful development for contraception^25^. It has been postulated that in comparison to the progestogens used previously, NOMAC is better able to maintain endometrial stability in combination with E2, due to its low impact on endometrial oestradiol metabolism, allowing the maintenance of adequate endometrial E2 concentrations and thus preventing endometrial breakdown^29^.

The incidences of unscheduled bleeding/spotting observed in this trial for DRSP/EE were higher than observed in trials reported previously. In one study, unscheduled bleeding after cycle 6 was reported at 8.8%^32^, and in another study, an incidence of 5.4% was reported^34^. A possible explanation for the observed higher incidence of unscheduled bleeding/spotting is the use of the more accurate electronic diaries in the current study instead of paper diary cards or a woman's personal recollection during clinic visits^32^,^34^,^35^. In the current study, women could only enter the daily bleeding information retrospectively for the previous two days, providing very accurate values for the incidence of unscheduled bleeding/spotting, likely resulting in the higher incidences for DRSP/EE users than observed with paper diaries. In addition, slight differences in definitions and analytical methods might have contributed to the observed differences between contraceptives. Indeed, this issue was recently addressed by Mishell and coworkers^36^,^37^, who proposed standardised methods for data collection and analysis of vaginal bleeding pattern.

NOMAC/E2 exhibits a neutral to slightly positive effect on acne, which is consistent with its moderate effect on sex hormone-binding globulin (SHBG) and androgen levels^19^. DRSP/EE has been observed to show some efficacy in the treatment of mild-to-moderate acne vulgaris^36^-^39^, which may be related to the stronger induction of SHBG and more profound reduction of androgen production associated with the administration of 30 μg EE^19^. The more frequently reported acne as an AE can be largely explained by the trial methodology as the condition was assessed at each clinic visit and study doctors were obliged to report any worsening as an AE. This procedure deviates from the spontaneous, unsolicited way of AE reporting usually applied in clinical trials. Moreover, the frequent assessments may also have caused increased awareness of acne with both patients and doctors, which in itself has the potential to increase reporting rates.

Subjects on NOMAC/E2 experienced a 1.00 kg increase in mean body weight, compared to 0.35 kg for users of DRSP/EE. From placebo-controlled studies in typical contraceptive user populations it appears that women aged 18–35 years, who do not use hormonal contraception, gain on average 0.6–0.8 kg of body weight over a period of 6–9 months^40^-^42^, which is close to the change reported here for NOMAC/E2 over a period of one year. Indeed a recent extensive overview of the available clinical data has not found a causal relationship between hormonal contraceptives and body weight^43^. The increase of 0.35 kg reported for DRSP/EE over one year of treatment could be explained by the antimineralocorticoid action of DRSP, which results in some loss of body water in the first months of treatment^38^-^44^-^45^.

NOMAC/E2 was well tolerated and showed an AE profile largely similar to the comparator DRSP/EE, except for the reporting of acne, irregular withdrawal bleeding and body weight gain, and discontinuation related to these conditions. As discussed above, the incidences of these events may have been influenced by the frequently scheduled active assessments of acne, body weight and bleeding pattern, and the trial methodology related to AE capture. Differences between the groups may reflect differences in the pharmacological properties of the hormones as well as regimen, while the open-label design of the trial may have had some effect considering that DRSP/EE (3 mg/30 μg) is a well-established COC with a known positive effect on both acne and weight gain. While DRSP/EE shows some additional pharmacological effects on acne and body weight, NOMAC/E2 seems neutral, namely, it appears to have no effect on body weight and it is not a cure for acne. A similar neutral effect of NOMAC/E2 has been observed with respect to parameters of haemostasis,lipid metabolism, carbohydrate metabolism, carrier proteins, and surrogate markers of adrenal and thyroid function^46^,^47^. However, long-term clinical studies with the appropriate clinical endpoints will be required to assess the clinical relevance of these observations.

Overall,the 24/4 monophasic regimen of NOMAC/E2 showed strong contraceptive efficacy and an acceptable cycle control with short and light withdrawal bleeding when administered for 13 cycles in over 1500 women. NOMAC/E2 was well tolerated and showed a safety profile that was similar to DRSP/EE.
